# Comparing Perfusion Index and Visual Analogue Scores for Postoperative Pain Assessment Following Upper Limb Surgeries Under Supraclavicular Brachial Plexus Block: An Observational Study

**DOI:** 10.7759/cureus.55529

**Published:** 2024-03-04

**Authors:** Pooja Bihani, Akanksha Pandey, Manish Jha, Naveen Paliwal, Rishabh Jaju, Rajendra Solanki

**Affiliations:** 1 Anesthesiology, Dr. Sampurnanand Medical College, Jodhpur, IND; 2 Anesthesiology, Pacific Medical College and Hospital, Udaipur, IND; 3 Anesthesiology, All India Institute of Medical Sciences, Deoghar, Deoghar, IND

**Keywords:** analgesia, visual analog scale (vas), pain, objective measure, perfusion index

## Abstract

Background: Effective pain assessment is crucial to tailor the analgesic regimen post-operatively. Perfusion index (PI) has been reported to be a useful objective assessment tool for monitoring pain. This study aimed to explore the utility of PI in assessing postoperative pain in upper limb surgeries under supraclavicular block and its correlation with visual analogue scale (VAS) scores.

Methods: This prospective, observational study included 140 patients scheduled for elective upper limb surgeries. PI, VAS scores, heart rate (HR), mean arterial pressure (MAP) and physiological parameters were recorded at baseline and postoperatively. Inj. tramadol was administered when the VAS score exceeded ≥ 4 and the VAS score, PI, HR and MAP were recorded at 5, 10, 15 and 20 minutes after administration. Comparison of normally and non-normally distributed data was done using t-statistics and Mann-Whitney U-test respectively. Pearson correlation was used to establish a correlation between variables and the receiver operating characteristic (ROC) curve was used to calculate the cut-off value of PI to determine the onset of pain.

Results: There was a significant and moderate correlation between pre-analgesic and post-analgesic PI and VAS score (r = -0.425 and -0.448 respectively, p<0.001), while PI and MAP or PI and HR showed only a weak correlation. A cut-off value of 14.7 for PI showed 76.3% sensitivity and 100% specificity in predicting rescue analgesia requirements.

Conclusion: The study supports the use of PI as an objective measure for postoperative pain assessment, with a notable correlation with VAS scores. The identified cut-off value for PI adds to its clinical utility in predicting the need for rescue analgesia.

## Introduction

Post-operative pain management remains an important concern for patients and pain assessment is another vital parameter to evaluate the intensity of pain, select the most appropriate analgesic and gauge response to treatment. Several subjective and semi-objective pain assessment scales such as the visual analogue scale (VAS) and numeric rating scale (NRS) for pain assessment have been used; however, objective assessment of pain assessment has now gained attention [1,2]. Few objective assessment tools such as the surgical stress index during anaesthesia and analgesic nociception index in the postanesthesia care unit have been used in past to supplement subjective assessment [3,4].

An emerging approach to pain assessment involves the use of the perfusion index (PI). PI is a metric that indirectly measures peripheral perfusion in a non-invasive and continuous manner, using pulse oximetry. It typically falls within the range of 0.02% to 20%. Pain-induced sympathetic-mediated vasoconstriction decreases PI and pain relief by a successful administration of regional anaesthesia often increases PI [5]. The VAS score is a widely utilized tool for assessing pain in postoperative patients, while PI is also recognized in the literature as a valuable metric for gauging pain severity in this demographic. The significance of PI as a tool for postoperative pain assessment lies in its capacity to complement subjective evaluations in patients who may face challenges in comprehending such as those with psychiatric conditions and older adults. Nevertheless, it’s still a work in progress as only limited research have examined the correlation between subjective VAS scores and objective PI measurements as indicators of postoperative pain assessment, even within the adult patient population [6,7]. This study, therefore, seeks to investigate the correlation between PI and VAS scores in assessing postoperative pain among adult patients undergoing upper limb surgery under supraclavicular block anaesthesia. The primary objective of the study was to see the correlation between PI and VAS scores. The secondary objectives were to determine cut-off values of PI to determine the onset of pain and to see the correlation of PI with physiological parameters such as mean arterial pressure (MAP) and heart rate (HR).

## Materials and methods

This prospective, observational study was performed after seeking clearance from the institutional ethical committee. Written and informed consent was obtained from patients to participate and possible dissemination of the study results for research purposes only. Consecutive patients of both sexes, aged between 18-65 years of age, and belonging to the American Society of Anesthesiologist (ASA) category I-II scheduled for elective upper limb surgeries (such as fracture radius, fracture both bone forearm, fracture lower end of humerus, contracture release below elbow and fracture metacarpals) under supraclavicular brachial plexus block were included in this study.

Patients, allergic to local anaesthetics and study drugs (ropivacaine, midazolam, tramadol and paracetamol), those with infection at the local site, pre-existing cardiovascular, pulmonary, peripheral vascular or metabolic diseases, bleeding disorders or with a history of seizures, neurological, psychiatric or chronic pain disorder on psychotropic drugs, were excluded from the study. We excluded patients who underwent upper limb surgeries (forearm, arm) using a tourniquet and those who had to be administered general anaesthesia (GA) due to inadequate block. Patients who required vasopressors or vasoconstrictors drugs in the postoperative period were considered drop-outs and excluded from data analysis.

A routine pre-anaesthesia checkup (PAC) was done for all patients one day prior to surgery VAS score was explained. Inside the operating theatre (OT), standard ASA monitors - electrocardiography (ECG), non-invasive blood pressure (NIBP) and pulse oximetry (Masimo SET® pulse oximeter; Irvine, California, USA) were attached and baseline vital parameters and PI recorded.

Ultrasound-guided supraclavicular brachial plexus block was performed by injecting 10-12 ml of 0.75% ropivacaine and surgery was started after achieving a successful block. It took 15-30 minutes to achieve a successful block. Midazolam bolus 1-1.5mg IV was given before performing the block for anxiolysis and additional 1 mg midazolam after 15 minutes was supplemented if the patient was still anxious. Post-surgery, patients were shifted to the recovery room and HR, NIBP and saturation were monitored continuously. Pain was measured subjectively by asking the patients to report their pain intensity on a VAS scale from 0 (no pain) to 10 (worst pain ever). At the respective time points, PI values were also obtained from the pulse oximeter probe attached to the index finger of the same upper limb, which underwent surgery. The time to first supplemental analgesia was determined as the duration from the participants' transition to the postoperative ward until they reported pain indicated by a VAS score exceeded ≥ 4. At this point, participants received 100mg IV tramadol in 100ml 0.9% normal saline over a duration of 15 minutes. The maximum allowable daily dose didn't exceed 400mg. We assessed VAS, PI and hemodynamic parameters 15 minutes after the completion of the infusion. If the patient still complained of pain, IV inj. 1 gm of paracetamol was administered as a supplemental analgesic administered and respective values of VAS, PI, HR and MAP were recorded.

Statistical analysis

The sample size was calculated using NCSS statistical software (NCSS, LLC, Kaysville, UT, USA) for power analysis and using the primary outcome variable i.e. the correlation between PI and VAS score [8]. Based on a study by Tapar et al. [7], considering an expected correlation coefficient of 0.255 and an alpha error of 5%, a sample size of 135 patients was calculated at a study power of 85% to detect statistical significance. Data were statistically described in terms of mean ± SD for normally distributed variables. Unpaired t-test and paired t-test were used for the comparison of normally distributed intergroup and paired data respectively and Mann-Whitney U-test was used for non-normally distributed variables. Correlation between variables before and after tramadol administration was analyzed using Pearson correlation analysis. The receiver operating characteristic (ROC) curve was plotted and the cut-off value of PI was determined from the curve. Data analysis was performed by Statistical Package for Social Sciences (SPSS) version 20.0 (IBM Corp., Armonk, NY, USA). A p-value < 0.05 was considered statistically significant.

## Results

**Figure 1:**
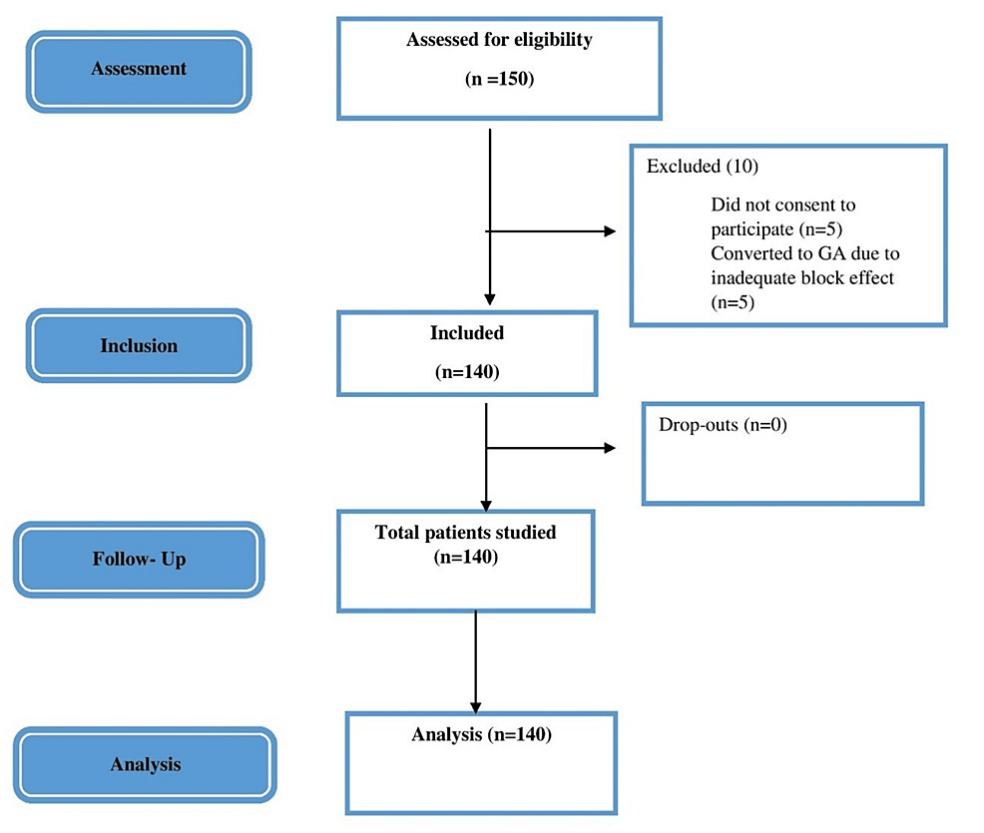
STrengthening the Reporting of OBservational studies in Epidemiology (STROBE) flow chart GA: General anaesthesia

A total number of 150 patients were recruited in the study as per inclusion criteria. Ten patients were excluded as five of those refused to participate and the other five had to be administered GA due to inadequate blockade. A total of 140 patients were included in the study for final analysis (Figure 1).

The mean age of patients was 44.2±8.2 years; 70% of whom were male and the rest 30% were females. The mean time to first supplemental analgesia was 5.8±2.2 hours post-operatively (Table 1).

**Table 1:** Demographic profile and time to first supplemental analgesia

Variable	Number (n=140)
Age; years (mean±SD)	44.2 ± 8.2
Gender; M/F (%)	98/42 (70/30)
Body mass index (kg/m^2^) (mean±SD)	23.32 ± 3.20
American Society of Anesthesiologists (ASA) grade; I/II (%)	71/69 (50.7/49.3)
Controlled Diabetics (%)	32 (22.85)
Controlled Hypertensives (%)	36 (25.71)
Smokers (%)	24 (17.14)
Time to first supplemental analgesia; hours (mean±SD)	5.8±2.2

Administration of analgesics resulted in a significant decrease in both HR and VAS scores and a concurrent increase in PI, while changes in MAP were statistically insignificant (Table 2).

**Table 2:** Perfusion index (PI), visual analogue scale (VAS), heart rate (HR) and mean arterial pressure (MAP) before and after analgesic administration

Variables	Pre-analgesic (mean±SD)	Post-analgesic (mean±SD)	P value
Perfusion Index (PI) (%)	11.09±3.53	14.93±3.19	<0.001
Visual Analogue Score (VAS)	6.07±0.87	2.32±0.97	<0.001
HR (beats per minute)	106.09±10.03	68.23±10.25	<0.001
MAP (mm. Hg.)	88.5±12.23	82.7±11.03	0.07

Pre-analgesic correlation between VAS and PI showed an inverse moderate correlation (r = -0.425, p<0.001). Similarly, after administration of analgesic, an inverse moderate correlation was seen between VAS and PI (r = -0.448, p<0.001). Pre-analgesic HR and PI showed a significant but weak correlation (r= -0.2, p<0.01) while post-analgesic HR and PI showed a significant but mild correlation (r=0.39, p<0.001). The pre-analgesic and post-analgesic correlation between MAP and PI were (r=0.06, p<0.45) and (r=-0.012, p<0.87) respectively, showing a very weak correlation between these two variables (Table 3).

**Table 3:** Correlation of perfusion index with visual analogue score, heart rate and mean arterial pressure VAS: Visual analogue score, PI: Perfusion index (%), HR: Heart rate (bpm), MAP: Mean arterial pressure (mm Hg)

Parameter		Correlation coefficient (r-value)	P value
VAS and PI	Pre-analgesic	-0.425	p<0.001
	Post-analgesic	-0.448	p<0.001
VAS and HR	Pre-analgesic	-0.2	p<0.01
	Post-analgesic	-0.39	p<0.001
VAS and MAP	Pre-analgesic	0.06	P=0.45
	Post-analgesic	-0.012	P=0.87

**Figure 2:**
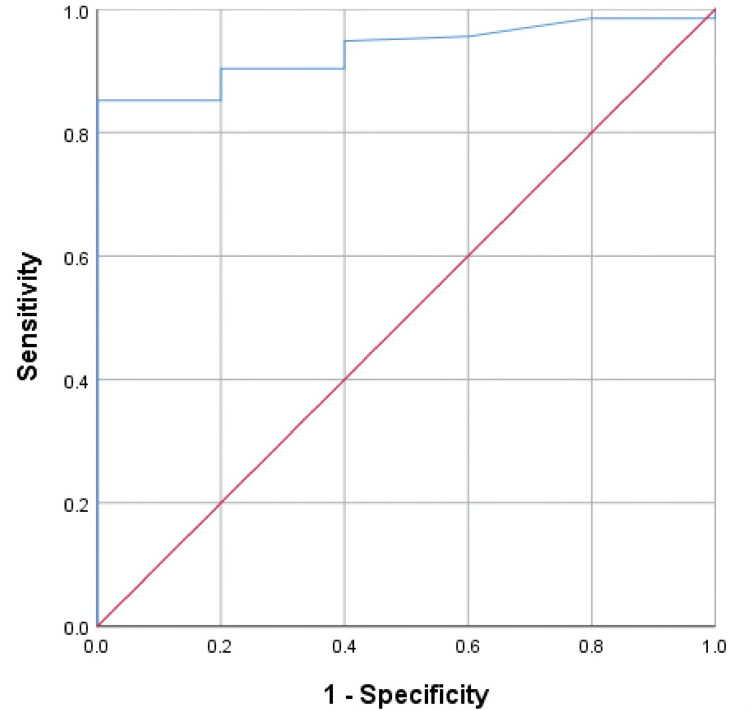
Receiver operating characteristic (ROC) curve

On ROC curve analysis, at a cut-off value of 14.7, PI could predict rescue analgesia requirement in the postoperative period with a sensitivity of 76.3% and a specificity of 100% (area under the curve (AUC) 0.933, 95% CI 0.878, 0.987) (Figure 2).

## Discussion

The principal findings of the study demonstrated that PI, VAS and hemodynamic variables such as HR and MAP show a significant difference after analgesic administration. There was a significant and moderate correlation between pre-analgesic and post-analgesic PI and VAS scores, while PI and MAP or PI and HR showed only a weak correlation. The cut-off value of PI for predicting rescue analgesic requirement was found to be 14.7 with a sensitivity of 76.3% and specificity of 100%.

PI is a non-invasive parameter derived from plethysmography, reflecting the ratio of pulsatile to non-pulsatile blood flow at the sensor site. It has been investigated for its potential as a real-time predictor of hypotension, an indicator of successful nerve block, a stress response marker during anaesthesia, and a marker of the depth of anaesthesia and recovery time in ICU [9-14].

Recent studies have also explored its role as an objective marker for pain intensity and effectiveness of analgesia [7,15]. Pain-induced sympathetic stimulation typically leads to peripheral vasoconstriction, resulting in a decrease in PI values. Conversely, the administration of analgesics tends to increase PI values and simultaneous decrease in VAS score and hemodynamic parameters. In this context, our study aimed to explore the correlation between PI and other pain assessment variables such as VAS scores and hemodynamic parameters.

In our study, a statistically significant and moderate inverse correlation was observed between VAS and PI both before and after analgesic administration. Studies on the utility of PI as an objective marker of postoperative pain, though have indicated its efficacy in predicting pain and assessing the adequacy of analgesia, a mild or no correlation has been observed between PI and VAS scores in literature. Gulen et al. investigated the use of PI as an objective marker for pain relief and the need for rescue analgesia in patients with renal colic in the emergency department and found PI to be a useful parameter for predicting requirements of rescue analgesia, with a weak correlation between VAS and PI before analgesic administration and no correlation after administration [16]. Another study found no correlation between PI-VAS values before and after analgesic administration in elective post-operative patients and a weak negative correlation between the difference of post- and pre-analgesic PI (PI-D) and the difference of post- and pre-analgesic VAS (VAS-D) [7].

The correlation observed between VAS and PI in our study suggests that PI can be a valuable tool for assessing acute post-operative pain after regional anaesthesia. The correlation between VAS and PI may vary across different patient populations and types of surgeries. Notably, the utility of PI becomes particularly evident in patients with cognitive dysfunction, elderly patients and those with psychiatric disorders who may face challenges with subjective self-reporting. Surekha et al. also conducted a study that suggested that PI could serve as a valuable tool for evaluating the severity of pain during laparoscopic surgeries under GA [17].

In terms of hemodynamic variables, we recorded a moderate yet significant correlation between PI and HR, while a weak and statistically non-significant correlation was observed between PI and MAP. Hasanin et al. reported significant differences in PI and hemodynamic parameters, such as MAP and HR, before and after positioning-induced pain in intensive care unit (ICU) patients [15]. However, it is essential to acknowledge that changes in post-anaesthesia MAP and HR post-anaesthesia are likely multifactorial and attribution solely to pain and their consequent efficacy in evaluating pain relief may be limited.

We noted a significant predictive ability of PI with a cut-off value of 14.7 for anticipating the need for supplemental analgesia in the postoperative period for orthopaedic patients undergoing upper limb surgeries under supraclavicular block. Other studies have similarly reported distinct cut-off values for PI or delta PI (the difference in PI before and after analgesic administration) through ROC curve analysis across different subgroups, including intraoperative analgesia, postoperative pain, and pain in critically ill patients [6,7,15]. The higher cut-off values observed in our study could potentially be attributed to the sympatholytic effects associated with supraclavicular block in the specific patient population undergoing upper limb surgeries.

Some of the notable limitations of our study are that we have restricted it to a specific subgroup of the healthy adult population undergoing upper limb surgeries. Including a more diverse patient population with different characteristics and surgical procedures would enhance the external validity of the study. PI as a pain measurement tool may complement VAS only in patients who are unable to comprehend. It may not be an ideal pain measurement tool for patients under GA, those on vasodilators or vasopressors during the intraoperative or postoperative period and in the paediatric population. Additionally, acknowledging that this is an observational study that highlights the potential for observer bias and subjective variations in pain reporting and bias related to study design are inherited. Future research efforts could benefit from incorporating randomized controlled trials or other study designs that mitigate observer bias and provide more robust evidence. Studies with large populations and different patient characteristics may be conducted to further emphasize the PI and VAS correlation.

## Conclusions

In summary, the study concludes that PI has a moderate correlation with VAS score for post-operative pain assessment in upper limb surgeries, and along with the established cutoff value, increases its clinical utility in predicting the necessity for postoperative supplemental analgesia. The findings encourage further exploration and incorporation of PI into post-operative pain management protocols and its utility, particularly in awake patients who are unable to comprehend.
